# Erythropoietin Increases Myelination in Oligodendrocytes: Gene Expression Profiling Reveals Early Induction of Genes Involved in Lipid Transport and Metabolism

**DOI:** 10.3389/fimmu.2017.01394

**Published:** 2017-10-26

**Authors:** Georgina Gyetvai, Trisha Hughes, Florence Wedmore, Cieron Roe, Lamia Heikal, Pietro Ghezzi, Manuela Mengozzi

**Affiliations:** ^1^Department of Clinical and Experimental Medicine, Brighton and Sussex Medical School, Brighton, United Kingdom

**Keywords:** central glia-4, microarrays, CD36, Pnlip, IGF-1, tissue-protective cytokines, repair, ERK1/2

## Abstract

Several studies have shown that erythropoietin (EPO) has neuroprotective or neuroreparative actions on diseases of the nervous system and that improves oligodendrocyte (OL) differentiation and myelination *in vivo* and *in vitro*. This study aims at investigating the early molecular mechanisms for the pro-myelinating action of EPO at the gene expression level. For this purpose, we used a differentiating OL precursor cell line, rat central glia-4 cells. Cells were differentiated or not, and then treated with EPO for 1 or 20 h. RNA was extracted and changes in the gene expression profile were assessed using microarray analysis. Experiments were performed in biological replicates of *n* = 4. Differentiation alone changed the expression of 11% of transcripts (2,663 out of 24,272), representing 2,436 genes, half of which were upregulated and half downregulated. At 20 h of treatment, EPO significantly affected the expression of 99 genes that were already regulated by differentiation and of 150 genes that were not influenced by differentiation alone. Analysis of the transcripts most upregulated by EPO identified several genes involved in lipid transport (e.g., *Cd36*) and lipid metabolism (*Ppargc1a/Pgc1alpha, Lpin1, Pnlip, Lpin2, Ppard, Plin2*) along with *Igf1* and *Igf2*, growth factors known for their pro-myelinating action. All these genes were only induced by EPO and not by differentiation alone, except for *Pnlip* which was highly induced by differentiation and augmented by EPO. Results were validated by quantitative PCR. These findings suggest that EPO might increase remyelination by inducing insulin-like growth factors and increasing lipid metabolism.

## Introduction

Myelination is essential for the proper functioning of the central nervous system (CNS). Oligodendrocyte (OL) damage and remyelination failure cause progressive neurological disability in chronic demyelinating diseases, including multiple sclerosis (MS), and also play a role in the pathogenesis of other neurological diseases, such as stroke, amyotrophic lateral sclerosis, and Alzheimer’s disease (1, 2).

Myelination occurs in development and continues postnatally. OL progenitor cells (OPCs) are present in the adult CNS; upon OL injury, OPCs contribute to OL regeneration and remyelination. However, in demyelinating diseases endogenous remyelination is insufficient to re-establish motor and cognitive performance. Absence of remyelination is often due to the inability of OPCs to differentiate and mature to produce myelin. Therapeutic agents that can stimulate OPCs to differentiate and remyelinate axons could improve neurological functions and clinical outcome in many pathologies (3, 4).

Erythropoietin (EPO) has neuroprotective and neuroreparative actions in many models of disease and injury of the nervous system (5); although recent evidence points to the role of the EPOR/CD131 heteromeric receptor in mediating EPO reparative functions (6), EPO can act also through the classical EPOR homodimer (7, 8). EPO induces neurogenesis and oligodendrogenesis (9, 10); it preserves myelin and increases myelin basic protein (MBP) expression in experimental models of demyelination and in white matter injury (11, 12). EPO increases the differentiation of OL precursors and the maturation of late stage OLs *in vitro* and *in vivo* (8, 10, 13).

Understanding the mechanism by which EPO acts on OLs might help identify therapeutic targets for remyelination.

The mechanisms that mediate differentiation and maturation of OLs are not completely understood. Studies have been hampered by the limited availability of high numbers of primary OPCs. Central Glia-4 (CG4) cells, an OL precursor permanent cell line derived from rat brain, can remyelinate axons *in vivo* (14). *In vitro*, in the absence of mitogens and serum, CG4 cells differentiate into MBP-expressing OLs within 48 h; then myelin oligodendrocyte glycoprotein (MOG), a marker of myelin deposition, is produced (15, 16). Therefore, CG4 are considered a good *in vitro* model of CNS myelination (17). We recently found that EPO increases myelin gene expression in differentiating CG4 cells; EPO promoted differentiation of OPCs into MBP- and MOG-positive OLs (8).

The aim of this study was to investigate the mechanisms mediating the pro-myelinating effects of EPO on CG4 cells. To this purpose, we analyzed the changes in the gene expression profile induced by EPO in differentiating cells at two time points, 1 and 20 h after EPO treatment, focusing on the transcriptional changes occurring during the OPC to OL transition. The results highlight an inducing effect of EPO on genes previously known to play a role in myelination, such as insulin-like growth factor-1 (*Igf1*), *Igf2*, protein tyrosine phosphatase receptor type E (*Ptpre*), as well as genes involved in lipid transport and metabolism, including fatty acid translocase (*Fat/Cd36*), peroxisome proliferator-activated receptor-gamma coactivator (*Ppargc1a*/*Pgc1alpha*), and pancreatic lipase (*Pnlip*) (18–24).

## Materials and Methods

### Cell Culture

The wild-type CG4 cell line, a rat OL precursor cell line originally obtained from primary cultures of bipotential oligodendrocyte-type-2-astrocytes (O-2A), was kindly donated by Huseyin Mehmet, Imperial College, London. CG4 cells, as primary O-2A cells, can differentiate into mature OLs by withdrawal of growth factors (bFGF and PDGF) and of B104 mitogens, or into astrocytes by addition of 20% fetal calf serum. Undifferentiated cells are bipolar; after 2 days of differentiation they acquire about 90% of multipolar morphology. Differentiated CG4 cells express myelin proteins, including MBP and MOG (15–17).

CG4 cells overexpressing EPOR (CG4-EPOR) were generated as reported in our previous study (8). Briefly, CG4-EPOR cells were obtained by transduction of CG4 cells with the mouse *EPOR* gene in a constitutive lentiviral vector, modified to include the *V5* epitope, the mouse encephalomyocarditis internal ribosome entry site (*IRES*) and the enhanced green fluorescent protein (*EGFP*) reporter. The expression of recombinant V5-tagged EPOR in transduced CG4 cells was verified by measuring by flow cytometry the EGFP reporter expression, and by immunoblotting with the anti-V5-tag mouse monoclonal antibody (Invitrogen), as described (8, 25).

CG4-EPOR cells were seeded in poly-l-ornithine-coated 6-well plates (320,000 cells in 4 ml GM per well). They were maintained at the precursor stage by culture in growth medium (GM), consisting of Dulbecco’s modified Eagle medium (DMEM) (Sigma-Aldrich) supplemented with biotin (10 ng/ml), bFGF (5 ng/ml), PDGF (1 ng/ml), N1 supplement (all from Sigma-Aldrich), and 30% B104-conditioned medium, obtained as previously reported (8). After overnight culture, the cells were induced to differentiate into OLs by switching to differentiation medium (DM), consisting of DMEM-F12 (Invitrogen) supplemented with progesterone (3 ng/ml), putrescine (5 µg/ml), sodium selenite (4 ng/ml), insulin (12.5 µg/ml), transferrin (50 µg/ml), biotin (10 ng/ml), thyroxine (0.4 µg/ml), and glucose (3 g/l) (all from Sigma-Aldrich), as reported (8). After 3 h, some of the cells were treated with recombinant human erythropoietin (rhEPO; Creative Dynamics) at 10 ng/ml and cultured for 1 or 20 h prior to RNA extraction. There is extensive biological cross-reactivity between human EPO and the EPOs of other mammals. Human EPO is approximately 80% homologous to rodent EPO, and it has been shown to be biologically active in rodents for erythropoietic and neurotrophic functions (26, 27). Undifferentiated cells were seeded as above and cultured in GM without switching to DM for the whole length of the experiment.

To measure ERK1/2 phosphorylation by western blot, cells were plated in poly-ornithine coated 24 well plates at 200,000/well in GM for 24 h, then switched to DM and incubated overnight to starve them of growth factors present in GM. Cells were then preincubated with PD184352 (Cell Signaling, #12147) or DMSO for 1 h, then treated with or without EPO at 10 ng/ml for the indicated times. PD184352 was dissolved in DMSO at 25 mg/ml and then diluted in DM at the indicated concentrations.

### RNA Extraction

Each sample was lysed with 1 ml QIAzol (QIAGEN). Total RNA was extracted by using the miRNeasy system and protocol (QIAGEN). RNA purity and integrity were determined using a NanoDrop ND-1000 (NanoDrop Technologies) and an Agilent 2100 Bioanalyzer (Agilent Technologies). All samples had a A260/A280 ratio >1.8 and RNA Integrity Number >9.

### Microarrays

All experimental conditions were performed in quadruplicate; undifferentiated cells were cultured in quadruplicate but only three random samples were used for microarray analysis and all of the four samples for quantitative PCR (qPCR) validation. In total, 19 arrays were completed: 3 undifferentiated (und) and 16 differentiated: 4 differentiated (ctr) and 4 differentiated + EPO (EPO) at each time point (4 and 23 h of differentiation; 1 and 20 h after EPO treatment, respectively).

RNA was amplified, labeled, and hybridized onto Single Color SurePrint G3 Rat GE 8 × 60K Microarrays (AMADID:028279; Agilent) at Oxford Gene Technology, Oxford, UK. Following hybridization, the arrays were scanned to derive the array images. Feature extraction software v10.7.3.1 was used to generate the array data from the images.

### Microarray Data Analysis

Raw data in standard format from the microarray experiment have been deposited in the Gene Expression Omnibus (GEO) database of NCBI (28) and are accessible through GEO Series accession number GSE84687.^1^ Raw data were normalized and analyzed using GeneSpring (Agilent) and Excel software. Transcript expression levels between the experimental groups were compared by Student’s *t*-test done on the log_2_ of the gProcessed Signal, obtaining uncorrected *p*-values. Subsequent multiple comparison corrections were performed using the Benjamini-Hochberg False Discovery Rate procedure, obtaining adjusted *p-*values. Fold change in the expression was calculated as the ratio between the average of the gProcessed Signals of the various groups and expressed as log_2_. Differences in expression with an adjusted *p*-value < 0.05 and an absolute fold change ≥ 1.5 (log_2_ fold change ≥ 0.58) were considered statistically significant. Functional annotation and biological term enrichment was done using the Database for Annotation, Visualization, and Integrated Discovery (DAVID)^2^ (29). DAVID calculates a modified Fisher’s exact *p*-value to demonstrate enrichment. Categories with *p*-value < 0.05 were considered significantly enriched.

### Microarray Data Validation by RT-qPCR

Reverse transcription (RT) and real-time qPCR were carried out as reported (30) on total RNA from quadruplicate samples, using TaqMan^®^ gene expression assays (Applied Biosystems/Life Technologies) and Brilliant III qPCR master mix (Stratagene/Agilent Technologies). Gene expression was quantified using the ΔΔCt method, according to Applied Biosystems’ guidelines. Results were normalized to HPRT1 expression (reference gene) and expressed as log_2_ of the relative expression (ratio) vs one of the control samples (as indicated), chosen as the calibrator.

### Western Blot Analysis

Cells were lysed in RIPA buffer (Thermo Fisher Scientific) and total cellular extracts were incubated on ice for 30 min and then cleared by centrifugation (15,000 *g* for 20 min at 4°C). Protein concentration was measured with the BCA kit (Pierce) and 30 µg of cellular proteins were analyzed by 10% sodium dodecyl sulfate-polyacrylamide gel electrophoresis and transferred to a nitrocellulose membrane (Millipore) by electroblotting. Membranes were blocked for 1 h with 5% bovine serum albumin (BSA, Sigma-Aldrich) in TBS and then probed with rabbit polyclonal anti-phospho-ERK1/2 (Cell Signaling, #9101) followed by detection with goat-anti-rabbit-IgG-horseradish peroxidase conjugate (Sigma-Aldrich, #A0545), all in 5% BSA in TBS with 0.1% Tween 20 (Sigma-Aldrich). Membranes were then stripped with Re-Blot Plus Strong Solution (Millipore) and then blocked and reprobed with rabbit polyclonal anti-ERK1/2 (Cell Signaling, #9102). Protein bands were visualized using ECL detection reagent (GE Healthcare) and exposing the membranes to autoradiography films (Hyperfilms, GE Healthcare).

## Results

### Experimental Design

Undifferentiated CG4 cells cultured for 1 day in GM were switched to DM and treated with EPO (10 ng/ml) after 3 h; gene expression analysis was performed 1 and 20 h upon EPO stimulation (at 4 and 23 h of differentiation, respectively). The dose of EPO was chosen based on dose–response experiments published in our previous study; since in these cells EPO dose-dependently induced MOG and MBP expression with maximal induction at 8 ng/ml and no further increase at 80 and 400 ng/ml, the dose of 10 ng/ml was used throughout this study (8). EPO-treated were compared with untreated differentiating cells; the effect of differentiation was investigated by comparison with undifferentiated cells cultured for 1 day in GM in the same experimental conditions. Genes differentially expressed were identified by setting a cutoff fold change (FC) of 1.5 (log_2_ FC = 0.58) and cutoff *p*-value < 0.05 after applying the Benjamini–Hochberg (BH) correction for multiple tests.

### Genes Regulated by Differentiation and EPO

**Figure 1:**
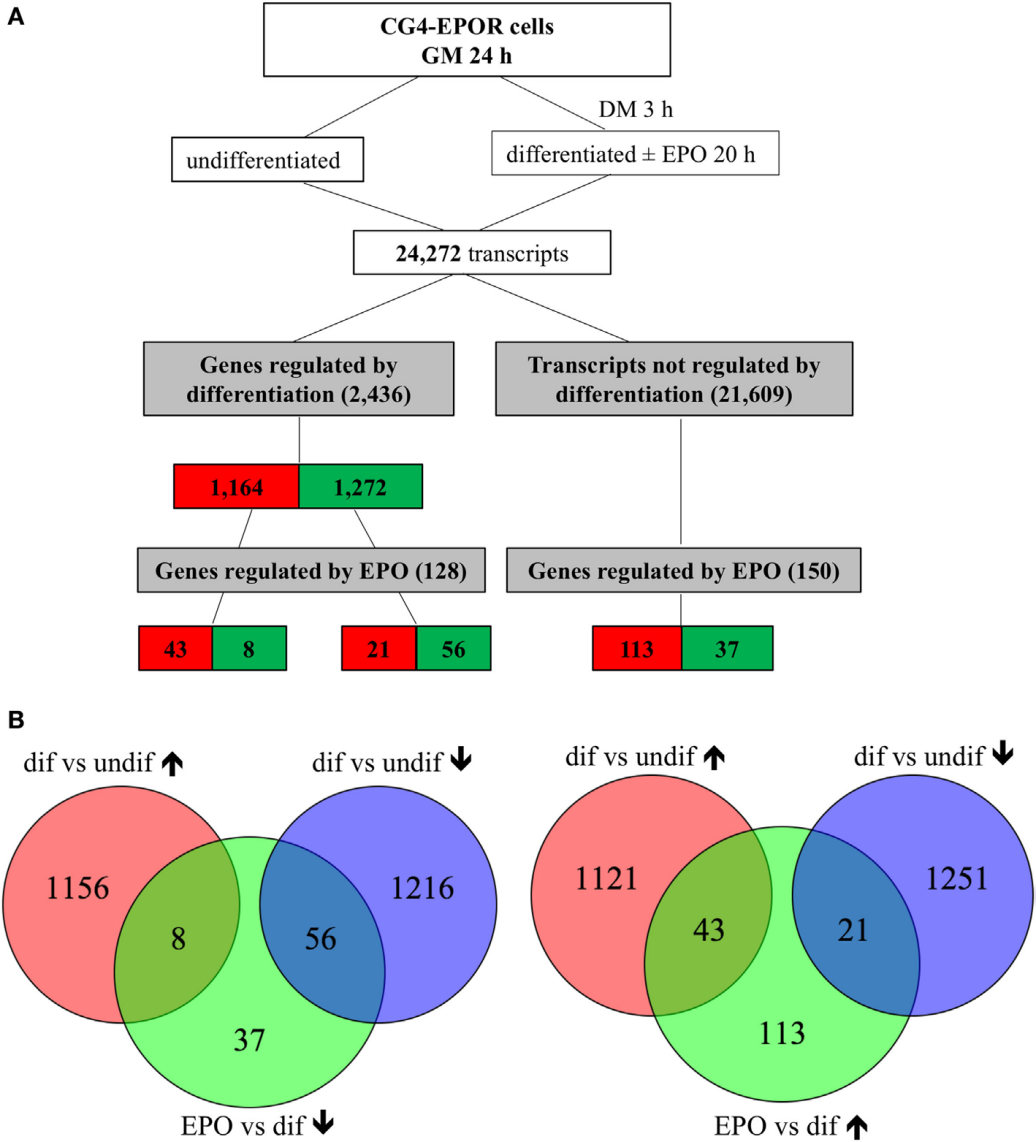
Transcripts regulated by erythropoietin (EPO) at 20 h in differentiating central glia-4 (CG4) cells. Cells cultured for 1 day in growth medium (GM) were switched to differentiation medium (DM); after 3 h, EPO was added and cells were incubated for further 20 h. **(A)** Transcripts regulated by differentiation were selected by comparing differentiating (23 h culture with DM) vs undifferentiated cells and transcripts regulated by EPO by comparing EPO-treated (20 h) vs untreated differentiating cells. Cutoff for selection was a fold change of 1.5 and BH adj. *p*-value < 0.05. The number of transcripts resulting from filtering is indicated and color coded (red, increased; green, decreased). **(B)** Venn diagrams representing the overlap between transcripts regulated by differentiation and downregulated (left diagrams) or upregulated (right diagrams) by EPO, selected as described in **(A)**.

At 20 h, culture in DM changed the expression of 2,663 out of 24,272 transcripts. Five genes represented by duplicate probes changed in opposite directions were excluded from the analysis; when genes were represented by duplicate or triplicates probes consistently changed in the same direction, only the most significantly changed one was included, obtaining 2,436 genes, of which 99 were further changed in the same direction by EPO; 21,609 transcripts were unaffected by differentiation, of which 174, representing 150 genes, were changed by EPO (Figure 1). The relative expression changes of all genes affected by differentiation (2,436; 1,164 upregulated and 1,272 downregulated; Figure 1) and/or by EPO (278; 177 upregulated and 101 downregulated; Figure 1) and details about the redundant probes removed are reported in Files S1–S3 in Supplementary Material.

On the other hand, 1 h of EPO treatment had little effect on the gene expression profile. Culture for 4 h in DM affected 931 transcripts out of 24,272; after removing one gene represented by duplicate probes changed in opposite directions, and redundant probes, 878 genes were obtained; of these, 461 were upregulated and 417 downregulated. The addition of EPO for the last hour increased the expression of only six transcripts, corresponding to five genes (*Egr1, H19, Fos, Arl4a, Irx2*), of which three (*Egr1, Fos, and Irx2*) had been decreased, and therefore changed in the opposite direction, by previous culture in DM; the remaining two, *H19* and *Arl4a*, had been unaffected by differentiation. The experimental design flowchart at 1 h is shown in Figure S1 in Supplementary Material and the whole list of the genes affected by differentiation and/or by EPO at 1 h, with details about the removed probes, is provided in Files S4 and S5 in Supplementary Material.

To understand the general function of the genes up- or downregulated in differentiating cells at 20 h, we used DAVID to identify the overrepresented GO categories with biological process (BP) designation (GO:BP).

Strongly proliferating OPCs need to exit the cell cycle to start differentiating and producing myelin proteins (31). Therefore, unsurprisingly, the top overrepresented GO:BP categories identified by DAVID analysis of the 1,272 genes downregulated by differentiation (full list, File S1 in Supplementary Material) included “cell division” and “mitotic nuclear division.” Thus, differentiation inhibited the expression of many genes driving cell proliferation; this confirms previous studies in CG4 cells (15, 32) and in primary OLs (33, 34).

Erythropoietin further downregulated the expression of 56 genes already decreased by differentiation, and of 37 of the unaffected ones (Figure 1; File S3 in Supplementary Material); the top enriched GO:BP terms in both groups of genes included “DNA replication”; only 8 transcripts were downregulated by EPO but upregulated by differentiation, and a PubMed search did not highlight any link with OLs or myelination. Therefore, analysis of the EPO-downregulated genes revealed a strong inhibitory action of EPO on a network of genes involved in cell proliferation, some of which were already affected by differentiation; however, it did not suggest any other mechanism that might be triggered by EPO through gene downregulation. The DAVID analysis of the genes downregulated by differentiation and/or by EPO is reported in File S6 in Supplementary Material.

Among the 1,164 genes upregulated by differentiation alone (full list, File S1 in Supplementary Material), the top enriched GO:BP categories were “nervous system development” (*n* = 25), which included genes involved in myelination, such as *Mag* and *Erbb2* (35), “cell adhesion” (*n* = 30), including genes previously found upregulated in differentiating OLs, such as *Cd9, Neo1, Ninj2, Opcml, Tnr* (36), “fatty acid beta-oxidation” (*n* = 10), and “glycolysis” (*n* = 9), both required to provide intermediates for myelin synthesis and energy to support the myelination process (Table 1) (37, 38). EPO further upregulated 43 of the transcripts already increased by differentiation alone (Figure 1; File S2 in Supplementary Material), among which DAVID analysis did not highlight any enriched GO:BP category. However, by manual analysis we identified two myelin genes [*Mag and Pmp2*, a peripheral myelin protein which is also present in the CNS (39)], one gene belonging to the GO:BP “nervous system development” (*Mag*) and three genes to “cell adhesion” (*Cdhr2, Mag, Ntm*) but none to “fatty acid beta-oxidation” or “glycolysis.” Twenty-one transcripts were upregulated by EPO but downregulated by differentiation, including low density lipoprotein receptor (*Ldlr*), which is expressed in mature OLs (40) and myelin transcription factor 1 (*Myt1*), whose multiple functions on proliferation and differentiation of OLs, likely dependent on interacting molecules, have not been fully characterized (33).

**Table 1:** Top enriched functional GO:BP categories in transcripts upregulated by differentiation or specifically upregulated by EPO at 20 h.

GO:BP category	Fold enrichment	Gene symbols	*p*-value
**Upregulated by differentiation (1,164)**			
Nervous system development	2.7	*GRIP1, ERBB2, GPM6B, TNR, SMIM3, SH2B2, NDRG2, BHLHE40, CABLES1, SIM2, INA*, ***MAG***, *NR4A2, DPYSL3, CSRP1, PTPRO, GAS7, PURA, SLITRK1, NTRK1, VEGFA, OPHN1, IFT88, GFRA2, KCTD11*	2.2E−05

Cell adhesion	2.2	*CHE, OPCML, BCAR1, NINJ2, NEO1, CD9, IGSF11, LGALS3BP, PTK2, SORBS2, TNR, ACAN, EMB, NEGR1, MLLT4, SPON1*, ***MAG***, *ICAM5, PODXL*, ***CDHR2***, *ITGA4, CERCAM, SSPO, FARP2, VWF, LSAMP, CX3CR1, RELN, PDZD2*, ***NTM***	8.2E−05

Fatty acid beta-oxidation	4.8	*CPT1C, ACAA2, ACADSB, CPT2, EHHADH, ABCD2, DECR1, HSD17B4, HADH, ACAA1B*	1.7E−04

Glycolytic process	4.8	ALDOA, ALDOART2, TPI1, PFKL, ALDOC, ENO2, PFKM, PGK1, DHTKD1	4.3E−04

**Upregulated by erythropoietin (EPO) and unchanged by differentiation alone (113)**			
Response to organic cyclic compound	6.2	*PRKCQ, FOS, CYP1B1, PLIN2, CD44, CTGF, IGF-1, IGF-2, TIMP3, PPARGC1A*	3.1E−05

Response to activity	11.2	*PPARD, CD36, IGF-1, FGF21, ZEB1, PPARGC1A*	1.9E−04

Response to nutrient levels	10.1	*IGF-1, IGF-2, FGF21, ZEB1, PPARGC1A, H19*	3.1E−04

Positive regulation of ERK1 and ERK2 cascade	6.8	*SPRY2, CD36, CD44, CTGF, ANGPT1, FGF21, HTR2C*	5.5E−04

*DAVID Functional Annotation Chart Analysis showing the four top overrepresented GO:BP categories among the transcripts upregulated by differentiation or specifically upregulated by EPO and unchanged by differentiation alone. The fold enrichment and the significance of the enrichment (a modified Fisher’s exact *p*-value calculated by DAVID) is reported. In bold the genes further upregulated by EPO (Table S2 in Supplementary Material)*.

### Genes Specifically Induced by EPO

We then looked at the transcripts specifically increased by EPO but unchanged by differentiation alone (113; Figure 1). The 15 most induced genes in this group are reported in Table 2 (full list, File S2 in Supplementary Material). Some of these genes have been described to play a role in myelination, including *Igf1* and *Igf2* (21), and *Ptpre*, a protein tyrosine phospatase whose knockouts have defects in myelination (19). Interestingly, PTPRE also inhibits ERK activation (41). DAVID analysis identified enrichment of generic GO:BP terms including “response to organic cyclic compounds” (*n* = 10), “response to activity” (*n* = 6), “response to nutrient levels” (*n* = 6), and positive regulation of ERK1 and ERK2 cascade (*n* = 7) (Table 1).

**Table 2:** Top 15 transcripts upregulated by erythropoietin (EPO) in differentiating cells at 20 h.

ProbeName	GeneSymbol	GenbankAccession	EPO vs differentiation		
			Log_2_ FC	*p*-value	BH adj. *p*-value
A_44_P342289	*H19*	NR_027324	12.53	2.4E−07	3.2E−04
A_64_P054808	*Cd36*	NM_031561	6.98	5.4E−08	1.5E−04
A_44_P792784	*Htr2c*	NM_012765	5.14	4.2E−09	5.1E−05
A_64_P128810	*RGD1565355*	NM_001109218	5.11	2.7E−08	9.3E−05
A_64_P069419	*Tnfrsf11a*	NM_001271235	4.37	4.9E−09	4.0E−05
A_64_P137130	*Ptpre*	NM_053767	4.01	3.3E−07	3.5E−04
A_64_P092747	*Mrvi1*	NM_001105210	3.96	7.9E−06	2.7E−03
A_44_P1037953	*Igf2*	NM_031511	3.68	5.7E−08	1.4E−04
A_64_P093467	*Trpc4*	NM_080396	3.42	8.5E−07	7.4E−04
A_44_P1058692	*Angpt1*	NM_053546	3.41	3.0E-06	1.4E−03
A_64_P080817	*Cd44*	NM_012924	3.34	2.4E−06	1.2E−03
A_44_P577108	*Rasgef1c*	NM_001108273	3.08	1.1E−07	2.0E−04
A_64_P082924	*Rspo2*	XM_006241608	3.07	7.4E−06	2.7E−03
A_44_P366723	*Igf1*	NM_178866	2.96	1.4E−06	9.3E−04
A_64_P053785	*Adra2a*	NM_012739	2.89	1.0E−05	3.3E−03

*The top 15 genes significantly induced by EPO at 20 h and unchanged by differentiation alone are listed. Cutoff for significance: fold change (FC) > 1.5 (log_2_ FC > 0.58) and BH adj. *p*-value < 0.05 in EPO-treated differentiated cells vs differentiation alone. Gene name of RGD1565355: similar to fatty acid/translocase/CD36*.

### EPO Increases the Expression of *Cd36* and of Genes Involved in Lipid Metabolism

We noticed, in the list in Table 2, a strong effect of EPO (about 50-fold induction) on *Cd36*, which mediates long-chain fatty acid uptake and metabolism (22, 42).

We, therefore, searched manually for other genes involved in lipid transport and metabolism among all the transcripts increased by EPO, including those also changed by differentiation alone (177; Figure 1; File S2 in Supplementary Material). We specifically searched for genes annotated with GO:0006629 “lipid metabolic process,” GO:0006631 “fatty acid metabolism,” GO:0006635 “fatty acid beta-oxidation,” GO:0019395 “fatty acid oxidation,” and GO:0015909 “long-chain fatty acid transport.” Other than *Cd36*, our search identified *Ppargc1a*/*Pgc1alpha*, lipin 1 (*Lpin1*), *Lpin2, Pnlip*, peroxisome proliferator-activated receptor delta (*Ppard*), and perilipin 2 (*Plin2*). The effect of EPO on the expression of these genes in differentiating cells is reported in Table 3.

**Table 3:** Transcripts involved in lipid transport and metabolism induced by erythropoietin (EPO) at 20 h in differentiating cells.

ProbeName	GeneSymbol	GenbankAccession	EPO vs differentiation		
			Log_2_ FC	*p*-value	BH adj. *p*-value
A_64_P054808	*Cd36*	NM_031561	6.98	5.4E−08	1.5E−04
A_44_P305482	*Ppargc1a*	NM_031347	1.48	1.7E−04	1.6E−02
A_44_P191309	*Lpin1*	XM_006239912	1.00	2.9E−05	6.0E−03
A_44_P254984	*Pnlip*^a^	NM_013161	0.92	2.1E−05	5.2E−03
A_44_P1045748	*Lpin2*	NM_001108236	0.71	5.8E−04	2.9E−02
A_42_P458711	*Ppard*	NM_013141	0.67	1.3E−04	1.4E−02
A_42_P839964	*Plin2*	NM_001007144	0.67	1.3E−04	1.4E−02

*All the genes increased by EPO at 20 h identified with GO “lipid metabolic process” (GO:6629), “fatty acid metabolism” (GO:6631), “fatty acid oxidation” (GO:19395), “fatty acid beta-oxidation” (GO:6635), “long-chain fatty acid transport” (GO:15909) are listed. Cutoff for significance: fold change (FC) > 1.5 (log_2_ FC > 0.58) and BH adj. *p*-value < 0.05 in EPO-treated differentiated cells vs differentiation alone*.

*^a^Increased also by differentiation alone (log_2_ FC 1.9, *p*-value 9.7E−05, BH adj. *p*-value 3.7E−03 when comparing differentiated vs undifferentiated cells)*.

### Validation of Microarray Data by qPCR

**Figure 2:**
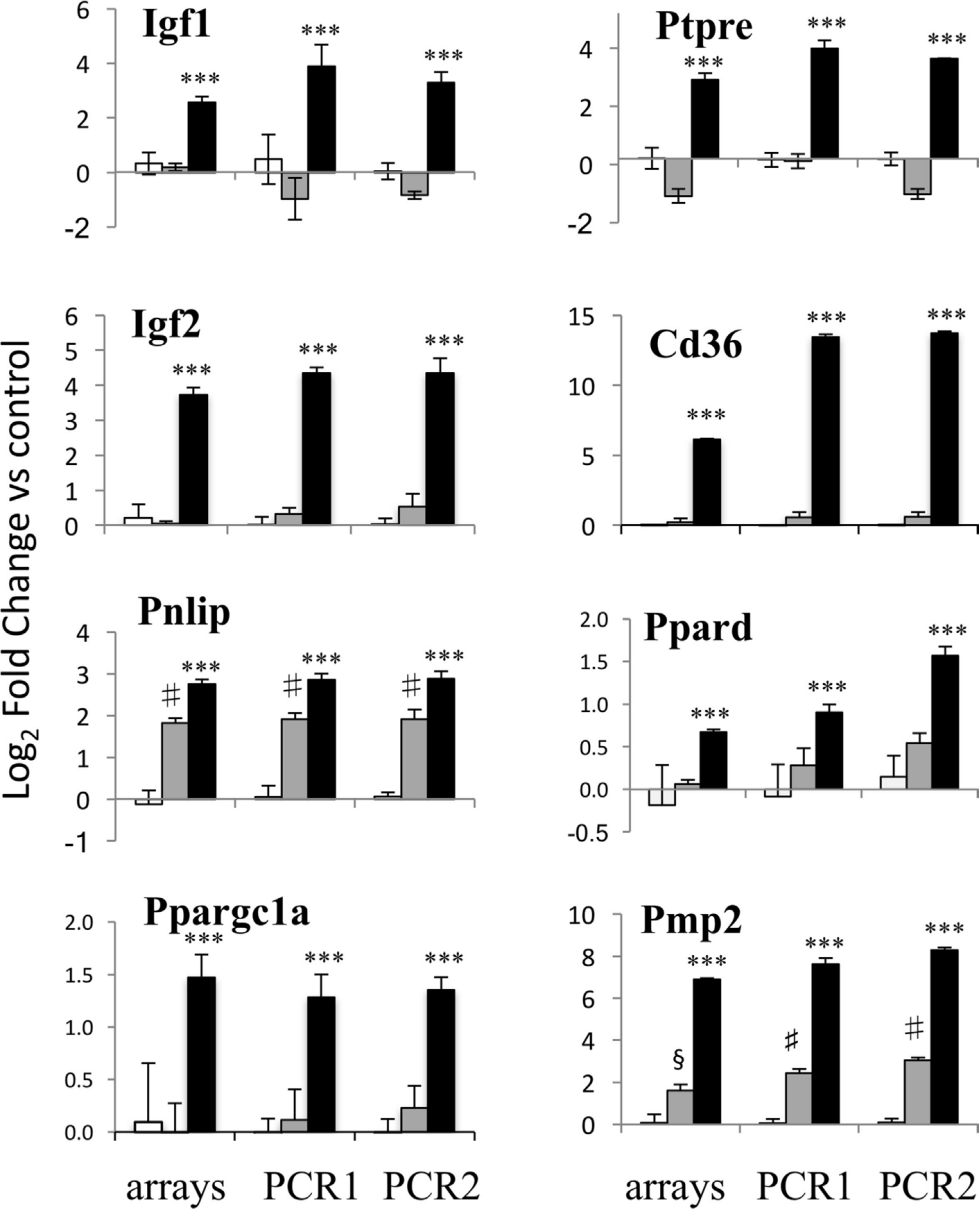
PCR validation of the microarray data. Results for eight genes at 20 h of erythropoietin (EPO) treatment are shown, comparing expression data from microarrays (left) with results from PCR analysis of the RNA from the same experiment (middle) and RNA from an independent experiment (right). Results for undifferentiated (white bars), differentiating (gray bars) and EPO-treated (black bars) differentiating samples are shown. Data are expressed as log_2_ fold change vs one of the respective undifferentiated samples and are the mean ± SD of four biological replicates. ***p < 0.001 vs differentiation alone; ^§^*p* < 0.01 vs undifferentiated; ^#^*p* < 0.001 undifferentiated by two-tailed Student’s *t*-test.

Microarray expression of eight genes of interest induced by EPO at 20 h, including the I*gfs, Ptpre*, some of the genes involved in lipid transport and metabolism and one myelin gene, *Pmp2*, was validated by RT-qPCR, using the same RNA used for the microarray experiment and RNA from a completely independent experiment (Figure 2). EPO-induced expression of all the eight genes tested was confirmed by RT-qPCR.

### An Inhibitor of ERK Potentiates EPO-Induced *Mog* Expression

**Figure 3:**
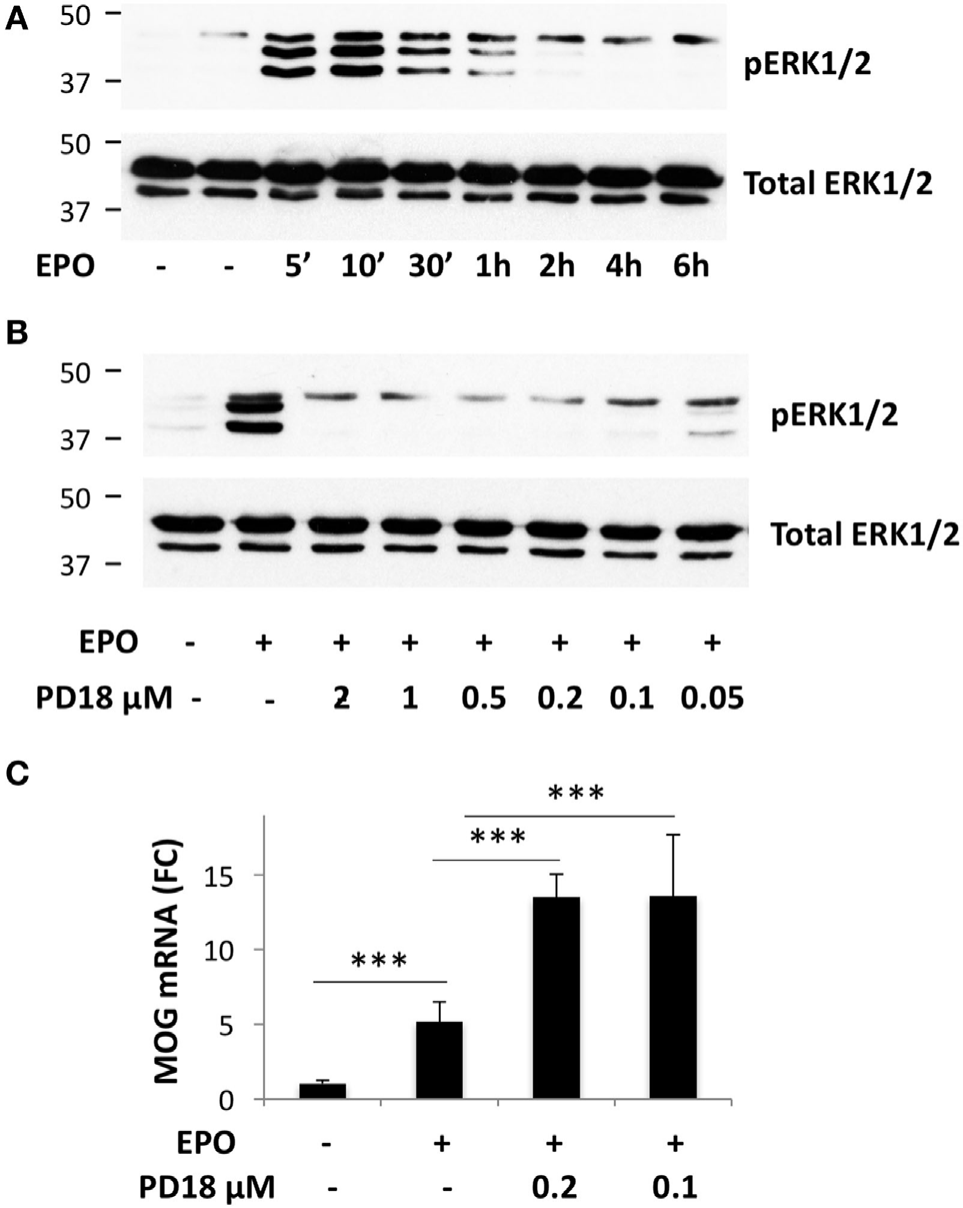
Inhibition of ERK1/2 phosphorylation potentiates erythropoietin (EPO)-induced *Mog* expression. **(A)** Time-course of EPO-induced phosphorylation of ERK1/2. **(B)** Dose–response of PD184352 on inhibition of ERK1/2 phosphorylation. **(A,B)** Cells were plated in GM for 24 h, then switched to DM and incubated overnight before treatment with medium alone or EPO at 10 ng/ml for the time indicated **(A)**, or treated with DMSO or with different concentrations of PD184352 for 1 h and then with EPO 10 ng/ml for 10 min. Phosphorylated ERK1/2 (upper bands) or total ERK1/2 as the loading control (lower bands) were analyzed by western blot. **(C)** PD184352 increases EPO-induced *Mog* expression. Cells were plated in GM for 24 h, then switched to DM and treated with DMSO or PD184352 for 1 h and then with EPO at 10 ng/ml. *Mog* expression was measured by RT-qPCR at day 3. Data are the mean ± SD of four biological replicates and are representative of three independent experiments. ***p < 0.001 by two-tailed Student’s *t*-test.

To investigate whether EPO-induced PTPRE might contribute to the increased myelin gene expression, possibly by inhibiting ERK1/2 activation, we used an inhibitor of ERK1/2 phosphorylation, PD184352. In cells incubated with DM for 1 day, EPO increased the phosphorylation of ERK1/2, which peaked at 10 min and returned to background level at 2 h (Figure 3A). Preincubation with PD184352 (0.1–2 µm) for 1 h completely inhibited EPO-induced ERK1/2 phosphorylation (Figure 3B), showing that the inhibitor was functionally active; PD184352 also potentiated EPO-induced *Mog* expression (Figure 3C). This result showed that inhibition of the phosphorylation of ERK1/2, achieved by EPO by inducing PTPRE, might be a mechanism by which EPO increases myelin gene expression.

## Discussion

The purpose of this study was to use gene expression profiling of CG4 OL cells to investigate the mechanism underlying the pro-myelinating action of EPO that we previously reported (8).

To this aim, we used CG4 cells transduced to overexpress EPOR. Evidence in the literature reports EPO effects on OLs, from cytoprotection to enhancement of differentiation and myelination, *in vitro* and *in vivo* (8, 10–13). EPOR is expressed in OLs in physiologic conditions, and its levels are increased by hypoxia, injury, and chronic disease (43, 44), suggesting the involvement of the EPO–EPOR pathway in remyelination upon injury and disease of the CNS. The low availability of high numbers of primary OPCs makes it difficult to study the mechanisms mediating EPO pro-myelinating effects. To circumvent this problem, we used the CG4 cell line, a well-documented *in vitro* model of CNS myelination (17). However, as reported in our previous study, wild-type CG4 cells do not express EPOR; in cells transduced to express EPOR, EPO increased myelin gene expression, and we demonstrated, using clones with differential EPOR expression, that the response to EPO was increased as the level of EPOR increased, and was actually due to expression of EPOR and not to the vector itself (8).

In our *in vitro* system, we observed an inducing effect of EPO on genes previously known to play a role in myelination, such as *Igf1, Igf2*, and *Ptpre*, as well as genes involved in lipid transport and metabolism, including *Fat/Cd36, Ppargc1a/Pgc1alpha*, and *Pnlip* (18–24).

In general, the changes in gene expression profile induced by EPO identified two patterns.

For 43 transcripts, EPO amplified the effect of differentiation, augmenting their induction. These include three of the genes identified by DAVID in the functional groups “nervous system differentiation” and “cell adhesion” (*Cdhr2, Mag, Ntm*). In addition, *Mag* and *Pnlip* were reported as upregulated in primary rat OL compared with OPC (20, 34, 36, 45).

A second group of genes was represented by the 113 transcripts upregulated by EPO and differentiation but not by differentiation alone. These include a number of genes identified in previous studies to be expressed in primary myelinating OL [*Acy3, Adamts4, Insc, Pdlim2, Prkcq* (46, 47)], suggesting EPO may support OPC differentiation. This helps to explain the mechanism, at the gene expression level, of the pro-differentiating action of EPO on OLs described in other studies (10, 12, 13).

Among the genes whose expression was induced by DM and EPO but not by differentiation alone, the *Igfs* (*Igf1* and *Igf2*) were among the top ones. IGF-1 effects on myelination *in vivo* and *in vitro* are well known (21, 48, 49). Of note, EPO-induced IGF-1 has been proposed as a mediator of EPO regenerative and remyelinating effects in the peripheral nervous system in rats (50). Therefore, IGF-1 might contribute to the effects of EPO in this system.

Both IGF-1 and IGF-2 are pro-myelinating cytokines, although the role of IGF-1 is better described (49). Interestingly, in the context of clinical samples, data in GEO^3^ (GSE38010) from Steinman’s group (51) show downregulation of *Igf2* in chronic MS plaques compared to healthy controls whereas *Igf1* expression did not change significantly.

Another gene among the top 15 induced by EPO was protein tyrosine phosphatase (PTP) receptor type E (*Ptpre*). Ranjan and Hudson found that PTP inhibitors decreased OL differentiation, and *Ptpre*, together with other PTP, was expressed in differentiating CG4 cells (18). Other PTPs have a role in OL maturation and myelination; *Ptpa* loss increased OPC proliferation (52); *Ptprz* knock out mice did not recover from EAE as well as controls and remyelination in MS plaques was associated with an upregulation of PTPRZ (53); also, hypomyelination was observed in *Ptpre* knock out mice (19).

A link between PTPRE and OL differentiation might be provided by its ability to inhibit the ERK pathway (41). The role of ERK in myelination is controversial; overall ERK activation increases myelination, for instance increasing myelin thickness (54). However, ERK also mediates cell proliferation induced by growth factors that maintain the OPCs in an undifferentiated state, including PDGF (55); of note, PTPRE inhibits PDGF signaling (56). In this context, since we are focusing on the early events necessary to drive myelination during OPC to OL transition, it is likely that ERK activation needs to be inhibited to inhibit OPC proliferation and promote OL differentiation. EPO-induced ERK1/2 activation in these cells, detected only at 10–60 min and then rapidly switched off, might be a negative feedback mechanism, not mediating but counteracting EPO-induced myelination; in this regard, we have previously shown that inhibiting *Egr2*, downstream to ERK, *Mog* expression is increased (8). Therefore, EPO-induced PTPRE, by inhibiting the ERK pathway, might favor OL differentiation.

Another pathway that seems a major target of EPO in differentiating OL is that of fatty acid transport and oxidation. *Fat/Cd36* was the second top induced gene. Of note, clinical data in GEO from Steinman’s group, mentioned above [see text footnote 3; GSE38010 (51)] report downregulation of *Cd36* in chronic MS plaques compared to healthy controls. The role of CD36 in mediating fatty acid uptake to enhance fatty acid oxidation has been described in muscle, heart, and adipose tissue (22, 42, 57); in macrophages, uptake of triacylglycerol-carrying lipoproteins *via* CD36 and lipolysis of triglycerides by lysosomal acid lipase increase oxidative phosphorylation of fatty acids and drive alternative (M2) macrophage activation (58). Together with *Cd36*, EPO increased the expression of *Pnlip*, recently shown to be expressed at high levels in differentiating OLs (20), possibly augmenting fatty acid uptake and utilization in OLs.

In addition, EPO-induced Ppargc1a/PGC-1alpha might mediate increased mitochondrial biogenesis; of note, EPO increases mitochondrial metabolism in heart and in muscle *via* PGC-1alpha (59, 60); moreover, both CD36 and PGC-1alpha can be induced *via* AMP kinase (AMPK) activation (61, 62), and EPO can activate AMPK in heart, in muscle and in white adipocytes (60, 63, 64). In the brain, EPO protection from ischemic damage is associated with preservation of *Pgc1alpha* expression (65); interestingly, PGC-1alpha is expressed in MBP-positive OL in the cerebellum and pharmacological upregulation of PGC-1alpha in OLs increased their differentiation, suggesting that PGC-1alpha has a role in CNS myelination (23, 66).

Altogether, increased expression of *Cd36, Pnlip, Pgc1alpha*, and other genes involved in lipid transport and metabolism (including *Ldlr*, not listed in Table 2 because inhibited by differentiation alone) suggests that EPO can increase lipid utilization in these cells; fatty acids might then be used for myelin synthesis. In support of this, supplementation of OL with polyunsaturated fatty acids, and in particular with gamma-linoleic acid, can increase OL differentiation and myelin gene expression (67).

It is also tempting to speculate that EPO might have the ability to induce a metabolic shift toward oxidative phosphorylation fueled by fatty acid oxidation, described in macrophages as a driver of M2 polarization (58, 68, 69), which might favor the differentiation of proliferating OPCs, mainly relying on aerobic glycolysis as a source of energy (38), into mature OLs. Although the brain mainly utilizes glucose as a source of energy, recent studies demonstrate that fatty acid oxidation can occur in neural and glial cells, including OLs (70–73). In addition, active mitochondrial metabolism has been recently described in mature OLs, previously thought to produce almost exclusively lactate to support neuron and axon survival (37, 74, 75). Interestingly, decreased oxidation of very long-chain fatty acids, reduced oxygen consumption and increased glycolysis have been described as mechanisms by which TNF inhibits OL differentiation (70, 76). Of note, dimethyl fumarate, a current effective therapy for recurrent MS, was found to increase oxidative metabolism and antioxidants levels and decrease the amount of lipids in OLs, suggesting that augmented lipid metabolism in OLs might mediate therapeutic effects in MS (77). It is interesting to note that PTPRE can also dephosphorylate insulin receptor (78), potentially inhibiting insulin signals and glucose utilization in these cells.

**Figure 4:**
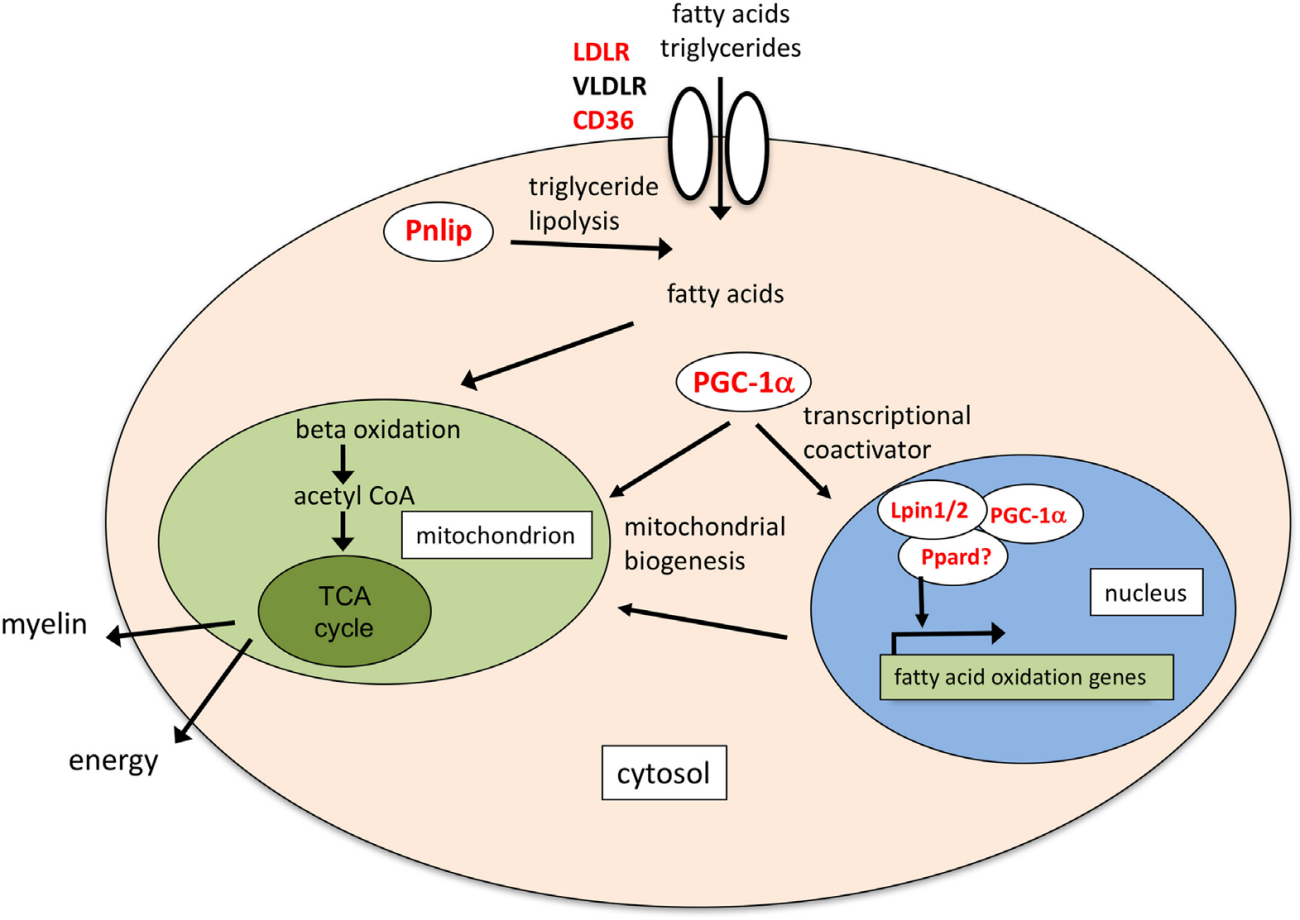
Schematic of the possible molecular functions of lipid transport and metabolism genes induced by erythropoietin (EPO). Pnlip hydrolyzes triglycerides into fatty acids (20, 34); CD36 mediates transport of long-chain fatty acids and triacylglycerol-carrying low density lipoproteins (22, 42). Together with low density lipoprotein receptor (LDLR) and very low density LR (VLDLR), expressed in mature OLs (40), it might provide a source of fatty acids to increase fatty acid oxidation. Ppargc1a/PGC-1alpha, Lpin1, and Ppard increase mitochondrial biogenesis and fatty acid oxidation (24, 81–83). In mouse liver, Lpin1 can act as a transcriptional coactivator interacting with PGC-1alpha and peroxisome proliferator-activated receptor alpha (Ppara) (81). Lpin1 can also interact with Ppard, which might, therefore, be part of the coactivation complex (84). Fatty acid oxidation might provide acetyl CoA for myelin synthesis, or increase ATP production through the TCA cycle. In red the genes induced by EPO.

In conclusion, we have identified genes specifically induced by EPO in differentiating OLs which might contribute to the myelinating effects of EPO; while IGF-1 had been reported as a mediator of EPO’s myelinating effects in the peripheral nervous system, increased EPO-induced *Igf1* and *Igf2* expression in OLs had never been described. As for the EPO inducing effect on PTPRE, it is interesting to note that another phosphatase, dual-specificity protein phosphatase 5 (*Dusp5*), was among the few genes induced by EPO in rats with cerebral ischemia, where EPO treatment is protective (30). Of note, DUSP5 and PTPRE can downregulate the ERK pathway (41, 79). An effect of EPO on PGC-1alpha has been described in other systems (59, 60, 65); associated with CD36, Pnlip and other genes involved in lipid metabolism, including Ppard, Lpin1, and Lpin2, might contribute to mediate EPO-induced myelination, as highlighted in Figure 4. Interestingly, the IGFs can increase fatty acid oxidation by upregulating CD36 (80). Further investigation is needed to establish whether these genes mapping to different pathways might separately contribute or work together to mediate EPO’s pro-myelinating effects.

A limitation of this study is that our results were obtained in an OL cell line in which EPOR was overexpressed. To generalize the relevance of our conclusions, our observations need to be confirmed in primary cells, including human cells, and eventually *in vivo*. In addition, the CG4 OL cell line is a model of CNS myelination, and therefore, our conclusions cannot be extended to the peripheral nervous system.

## Author Contributions

GG, TH, FW, CR, LH, and MM performed experiments; GG, MM, LH, and PG designed experiments; GG, TH, and MM analyzed data; MM, GG, TH, and PG wrote the manuscript; all authors critically revised and approved the manuscript.

## Conflict of Interest Statement

The authors declare that the research was conducted in the absence of any commercial or financial relationships that could be construed as a potential conflict of interest.

## Footnotes

^1^http://www.ncbi.nlm.nih.gov/geo.

^2^https://david-d.ncifcrf.gov.

^3^www.ncbi.nlm.nih.gov/geo.
