# Supplementary material for: Erythropoietin Increases Myelination in Oligodendrocytes: Gene Expression Profiling Reveals Early Induction of Genes Involved in Lipid Transport and Metabolism
Source: Front Immunol. 2017 Oct 26;8:1394. doi: 10.3389/fimmu.2017.01394 (PMC5662872; doi:10.3389/fimmu.2017.01394)
Supplement: Supplementary file 1 [file data_sheet_1.pdf]

Supplementary Material

EPO Increases Myelination in Oligodendrocytes: Gene Expression Profiling Reveals Early Induction of Genes Involved in Lipid Transport and Metabolism

Georgina Gyetvai, Trisha Hughes, Florence Wedmore, Cieron Roe, Lamia Heikal, Pietro Ghezzi, Manuela Mengozzi*

*Correspondence: Manuela Mengozzi m.mengozzi@bsms.ac.uk

Supplementary Material Contents:

Supplementary Figure 1, page 2

Supplementary Files S1-S6 Legends, page 3

Supplementary Files S1-S6 uploaded separately as excel files

1 Supplementary Figures

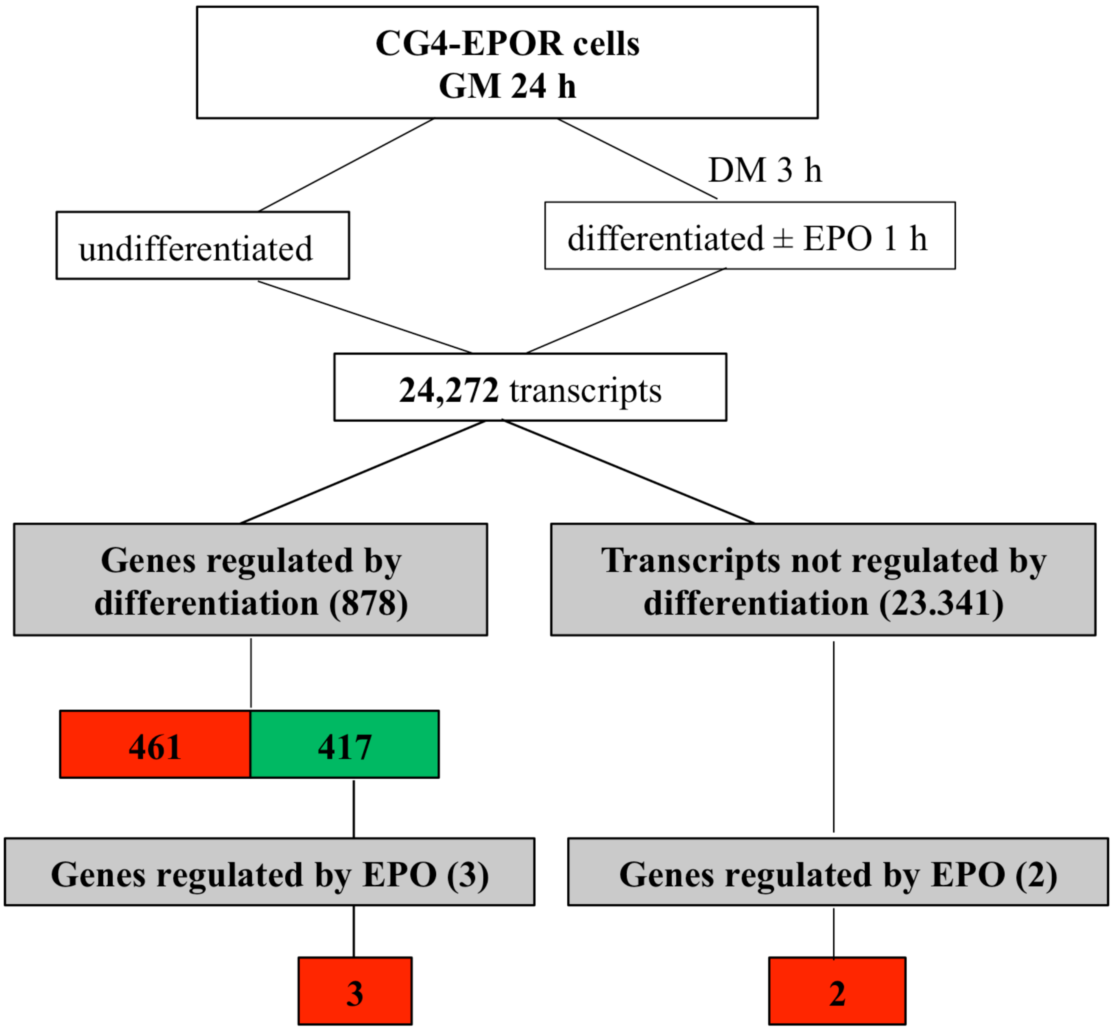

Supplementary Figure 1. Transcripts regulated by EPO at 1 h in differentiating CG4 cells. Cells cultured for one day in growth medium (GM) were switched to differentiation medium (DM); after 3 h EPO was added and cells were incubated for further 1 h. Transcripts regulated by differentiation were selected by comparing differentiating (4 h culture with DM) vs undifferentiated cells and transcripts regulated by EPO by comparing EPO-treated (1 h) vs untreated differentiating cells. Cut-off for selection was fold change of 1.5 and BH adj. p-value < 0.05. The number of transcripts resulting from filtering is indicated and colour coded (red, increased; green, decreased).

2 Supplementary Files Legends

File S1. **Genes significantly changed in differentiating cells at 20 h**. All genes changed more than 1.5-fold (absolute log_2 FC > 0.58), BH adj. p-value < 0.05 in differentiating (dif) cells at 20 h vs undifferentiated (undif) cells are listed. For genes represented by 2 probes (*) or by 3 probes (**) consistently changed in the same direction, only the most significanlty changed one is shown. Five genes significantly changed by differentiation (Gpr155, Ghrh, LOC68991, Sf3a1 and Tal1) but represented by duplicate probes changed in opposite directions were excluded from the analysis.

File S2. **Genes significantly upregulated by EPO in differentiating cells at 20 h and relative change in differentiating vs undifferentiated cells**. Only genes increased more than 1.5-fold (log_2 FC > 0.58), BH adj. p-value < 0.05 in EPO-treated vs untreated differentiating cells are listed; ns=not significant. For genes represented by 2 probes (*) consistently increased by EPO, only the most significanlty changed one is shown.

File S3. **Genes significantly downregulated by EPO in differentiating cells at 20 h and relative change in differentiating vs undifferentiated cells**. Only genes decreased more than 1.5-fold (log_2 FC < -0.58), BH adj. p-value < 0.05 in EPO-treated vs untreated differentiating cells are listed; ns=not significant. For genes represented by 2 probes (*) consistently decreased by EPO, only the most significanlty changed one is shown.

File S4. **Genes significantly changed in differentiating cells at 1 h.** All genes changed more than 1.5-fold (absolute log_2 FC > 0.58), BH adj. p-value < 0.05 in differentiating cells at 1 h vs undifferentiated cells are listed. For genes represented by 2 probes (*) or by 3 probes (**) consistently changed in the same direction, only the most significanlty changed one is shown. One gene significantly changed by differentiation (Camta1) but represented by duplicate probes changed in opposite directions was excluded from the analysis.

File S5. **Genes significantly changed by EPO at 1 h in differentiating cells and relative change in differentiating vs undifferentiated cells**. Only genes changed more than 1.5-fold (absolute log_2 FC > 0.58), BH adj. p-value < 0.05 in EPO-treated vs untreated differentiating cells are listed; ns=not significant. *Represented by 2 probes consistently increased by EPO of which only the most significantly changed one is shown.

File S6. Top enriched functional GO:BP categories of genes down-regulated by differentiation and/or by EPO at 20 h. DAVID Functional Annotation Chart Analysis showing the four top overrepresented GO:BP categories among the transcripts down-regulated by differentiation alone, further down-regulated by EPO, or specifically down-regulated by EPO and unchanged by differentiation alone. The fold enrichment and the significance of enrichment (p-value) is reported.
